# Ataxia patient care pathway and associated healthcare costs in Italy: a cross-sectional survey

**DOI:** 10.1007/s10072-026-09150-w

**Published:** 2026-07-16

**Authors:** Julie Vallortigara, Julie Greenfield, Barry Hunt, Alessandro Filla, Antonio Federico, Maria Litani, Deborah Hoffman, Steve Morris, Paola Giunti

**Affiliations:** 1 Ataxia Centre, Department of Clinical and Movement Neurosciences, UCL Queen Square Institute of Neurology, 9Th Floor, Queen Square House, Queen Square, London, WC1N 3BG UK; 2 Ataxia UK, London, UK; 3 Department of Neurosciences, Reproductive and Odontostomatological Sciences, Federico II University, Naples, Italy; 4 Department of Medicine, Surgery and Neurosciences, Medical School, University of Siena, Siena, Italy; 5 A.I.S.A Onlus, Sestri Levante, GE Italy; 6 Takeda Pharmaceuticals, Cambridge, MA USA; 7 Primary Care Unit, Department of Public Health and Primary Care, University of Cambridge, Cambridge, UK

**Keywords:** Ataxia, Care pathway, Costs, Health service use, Specialist centre

## Abstract

**Background:**

The ataxias are rare complex neurological disorders challenging for clinicians to manage. The costs of ataxia care and differences in healthcare use and treatment outcomes between specialised ataxia centres (SAC) and standard neurology services have not been investigated.

**Objective:**

This study explored patient pathways, healthcare use and costs, comparing SAC with non–specialist settings in Italy.

**Methods:**

A patient survey was distributed in Italy (May–September 2021) to collect information about the diagnosis and management of ataxias in SAC and non-specialist settings, healthcare service visits, and patients’ satisfaction. We compared per-patient mean resource use for each contact type and health service cost, stratifying patients by SAC attendance.

**Results:**

Among 174 participants, 44% saw a neurologist within 6 months of seeking medical advice, and 56% went to a SAC directly. Travelling was a top reason why people stopped going to a SAC and a barrier preventing patients from accessing it. SACs delivered better service to patients in various aspects of care. The differences in the number of contacts for various health services between the SAC and non-SAC groups were mostly non-significant. The mean total cost per patient over a one-year period was €1952 for non-SAC and €1666 for SAC.

**Conclusions:**

There was a trend towards patients’ appreciation for SAC services compared with non-SAC services, without significant change in costs. We identified difficulties in attendance and adherence despite the significant number of SACs in Italy. We believe that the combination of face-to-face and telemedicine could improve patients’ attendance at SACs.

**Supplementary Information:**

The online version contains supplementary material available at 10.1007/s10072-026-09150-w.

## Introduction

The ataxias are described as a group of rare, complex neurological disorders affecting primarily motor functions. Patients living with ataxia usually experience a lack of balance and coordination, slurred speech and impaired eye movements [1]. Additional symptoms associated with specific ataxias include spasticity, tremor, sensory disturbance, auditory and visual impairment, bladder and bowel dysfunction, cardiac complications, musculoskeletal complications, and cognitive impairment. Gait and balance problems often progress to the point at which patients become reliant on wheelchairs, and the level of disability increases at the cost of functional independence [2].

We focus on ataxias as disorders where ataxia is not an epiphenomenon of another neurological condition (e.g., multiple sclerosis) or due to other causes (e.g.: post-stroke ataxia). The possible diagnoses include inherited ataxias, autoimmune-mediated and idiopathic ataxias [1]. Over one hundred genetic ataxias have been described [3, 4]; however, global epidemiology references are scarce. Recent studies have estimated an ataxia prevalence rate of 26 cases per 100,000 individuals in children; in adults 2.7 cases per 100,000 individuals with hereditary dominant cerebellar ataxia (CA) and 3.3 cases per 100,000 individuals with recessive CA [5, 6]. Idiopathic degenerative ataxias have a prevalence rate of 2.2–12.4 per 100,000 [7]. Friedreich’s ataxia (FA), the most common inherited ataxia, has a prevalence of 0.59–2.1 per 100,00–0 in Italy [8].

A lack of awareness and understanding of ataxias among health care professionals (HCPs) makes their management challenging and suggests an important role for specialist clinical services for diagnosis and treatment [9–12]. Improving the time to reach diagnosis and early intervention for ataxia may contribute to reducing complications and disabilities. To date, there are no treatments available to modify the progression of most forms; however, some progress has been made in symptomatic interventions using holistic approaches with physiotherapy and occupational therapy (OT) [11–17]. Recently, a disease-modifying treatment for only FA, the Nrf2 activator Omaveloxolone, has been approved by the FDA and the EMA for people over 16 years of age [18, 19]. Ataxia management warrants a multidisciplinary approach [17]. Ataxia patients require complex care by a multidisciplinary team (MDT), including neurologists, geneticists, physiotherapists, OTs, speech and language therapists (SLTs), and neuropsychologists [11]. Specialist ataxia centres (SACs) can provide coordinated care and address the specific needs of ataxia patients [9, 10]. In Italy, SACs are based at University Hospital and/or are part of the IRCCS (Scientific Institute for Research, Care and Treatment).

This study evaluated the benefits of coordinated multidisciplinary care patterns on patients’ reported outcomes. This was part of a larger study surveying three European countries, the UK, Germany and Italy; the combined results of these countries are published [20, 21]. In this paper, we provide a more in-depth analysis of the care pathway of patients living with progressive ataxias in Italy. This project also studies the health economic effects of SAC compared with those of the usual care scenario in non-specialist settings. We conducted a survey to collect patients’ experience and feedback about the diagnosis and management of their ataxias together with the cost associated with attendance at SACs. We investigated the impact of comorbidities and the complexity of ataxia on resource use and costs. Ultimately, we aim to inform policy recommendations on how to improve treatment and care for people living in Italy with these complex neurological conditions.

## Methods

A focus group was created that included Italian patient organisation AISA (Associazone Italiana per la lotta alle Sindromi Atassiche) representatives and clinicians with expertise in ataxia based in Italy. At the time of our study, this group identified eleven SACs located at University Hospitals (Turin, Florence, Siena, Naples, Messina, Rome-Sapienza) or in IRCCSs (Scientific Institute for Research, Hospitalization and Healthcare, in Italian: Istituti di Ricovero e Cura a Carattere Scientifico) (Milan, Pisa, Genova, Bologna, Rome-Bambin Gesu) (Appendix 1).

### Patients

The population of interest was patients (or family members/carers as proxies) with ataxia, aged 16 years old or older (SACs are adults’ neurology services) and living in Italy. The survey was distributed by the AISA, with the support of the clinicians working at SACs, targeting individuals living with progressive ataxias, i.e., FA, inherited CA, sporadic or idiopathic CA.

### Survey design

The survey was designed and first piloted in the UK [22]. A working group with patient group representatives, a specialist ataxia neurologist, a specialist ataxia nurse, a health economist and representatives of pharmaceutical companies was involved in the design of this survey. The final version of the survey had 64 non-obligatory questions relating to the following topics: demographics, diagnosis, referrals, patients’ encounters with healthcare professionals, treatments received and patient satisfaction. After the pilot, both the Italian focus group, and a focus group in Germany [23], reviewed the survey; minor modifications were made including the addition of questions on mobility status and referral details to SACs and MDT clinics, and clarification of wording for cross cultural relevance as well as changing the lay out for some questions. The revised survey listing 47 non-obligatory questions was then translated and disseminated in Italy and in Germany [23]. The context of the study and the medical terms used were explained at the beginning of the survey (Appendix 1).

### Data collection

The survey was put online on the SurveyMonkey platform and rolled out during May–September 2021. The responses were then exported from SurveyMonkey and collated in Microsoft Excel for data cleaning and analysis. The data cleaning process involved the removal of all respondents who did not provide informed consent or positive responses to the two screening questions for an anonymised version of the database (questions 1 & 3; see Appendix 1). Where the respondents gave contradictory responses in different questions, the responses to those questions were removed from the analysis. Incomplete surveys, where some questions were left unanswered, were not removed from the analysis. The number of respondents was reported for each individual question.

### Ethics and consent

The survey was anonymised therefore no ethical approval was needed to roll out the survey in Italy. Consent was obtained online, with the act of submitting the survey considered as consent to take part in the study.

### Data analyses

Responses were stratified by attendance at SAC, comorbidities, and complexity of the disease For the attendance at a SAC, we used questions 25 “Have you ever been referred to/seen at one of the eleven specialist ataxia centres (Florence, Milan, Messina, Naples, Rome (2 centres), Siena, Turin, Pisa, Genova, Bologna)?”; and question 25i “If you have never been referred to/seen at a specialist ataxia centre, why is this?” (Appendix 1). People who had never been to a SAC were grouped in the ‘non-SAC’ group; people who are currently going to a SAC were grouped in ‘SAC’ group. There was a third group of people who used to go to a SAC but no longer attend; they were grouped in the ‘USED to SAC’ group. This group was not included in the statistical analysis because they experienced a mixed care pathway. However, the results of the survey for this group have been used to identify barriers to continuing to access SACs. For comorbidities, answers to question 6 “Do you have any additional conditions, not related to your ataxia?” (Appendix 1) were used for stratification. People who answered “none” were put in the group with no comorbidity on top of their ataxia. People who reported other conditions were put in the group with one or multiple comorbidities due to living with at least one other condition on top of their ataxia. For complexity of the disease, answers to question 36 “Have you ever been referred for treatment of any of the following symptoms as a result of your ataxia?” (Appendix 1) were used to create three groups. People who did not report any of the symptoms listed in that question were put in group 0. People who reported between one and four symptoms associated with their ataxia were put in group 1 (low level of complexity). People who reported between five and eight symptoms were put in group 2 (moderate level of complexity). In questions with responses measured on a Likert scale, the responses were grouped into two categories, where affirmative responses were considered to be ‘very positive/positive/slightly positive’ or ‘very effective/effective/slightly effective’ or ‘strongly agree/agree/slightly agree’ and negative responses were considered to be ‘very negative/negative/slightly negative’ or ‘very ineffective/ineffective/slightly ineffective’ or ‘strongly disagree/disagree/slightly disagree’. For questions 45 “Overall, including all symptoms mentioned above and any other symptoms that you experience as a result of your ataxia, do you feel your symptoms are well managed?” and 46 “How well do you feel your care reflects your needs?” (Appendix 1), affirmative responses were considered to be ‘best it could be/very well/quite well/adequately’. Results were prepared as tabulated descriptive statistics and presented as numbers (n) and percentage (%) of total respondents per question. The questions were treated independently, and the Chi-Square Pearson test was performed to compare results between the ‘SAC’ and ‘non-SAC’ groups for each question.

### Resource utilisation and costs

The survey was designed to capture patient‑reported healthcare resource use rather than the full economic burden of ataxia. The costs reported in this study are defined as direct healthcare costs only. Participants were asked to record the number of health care contacts they had received in the preceding 12 months, specifically due to their ataxia. Participants were asked to record the number of contacts with the family doctor, visits to see a resident neurologist/outpatient visits to a neurologist in a hospital with a general neurological department, SAC visits, hospital inpatient stays, emergency room visits, physiotherapy appointments, speech and language therapy appointments, occupational therapy appointments, and other consultant specialist visits (e.g. an ophthalmologist, an ear, nose and throat specialist, urologist, gastroenterologist and others). Unit costs for each type of contact were obtained from local providers or published sources [24]. All costs were calculated in 2023 € using GDP Purchasing Power Parities [25]. We compared average resource use for each contact type, and costs, over a 12- month period, stratifying patients by whether they were currently attending a SAC or had never attended a SAC. We then repeated this analysis, additionally stratifying by the presence of comorbidities (no/yes) and number of symptoms experienced as a result of ataxia (0, 1–4, 5–8 symptoms). We tested for significant differences in mean resource use and costs between the SAC/non-SAC groups using unadjusted and adjusted ordinary least squares regression analysis, the latter controlling for age, sex, plus the number of symptoms experienced as a result of ataxia and whether or not the patient had comorbidities, as appropriate. We also tested for significant differences in mean contacts and costs between sub-groups of patients by the presence of comorbidities and number of symptoms experienced separately for non-SAC and SAC groups using adjusted ordinary least squares regression analysis.

### Patient travel

As part of the survey, we also asked respondents who reported using the SAC to record the main mode of transport they used to travel to the centre, as well as the travel time from home to the centre. We also asked all respondents how long it took to travel one-way to receive ataxia care by a neurologist based in a general neurology clinic (not at a SAC) and the mode of transport they used. We present these data separately according to whether patients reported they were currently attending a SAC or had never attended a SAC.

## Results

### Demographics

The characteristics of the cohort are summarised in Table 1, including the proportion of groups based on SAC attendance. Most of the participants reported that ataxia affected their mobility: 57 (32.7%) used a wheelchair all the time, 28 (16.1%) used a wheelchair when outside, and 43 (24.7%) experienced moderate impairment and were able to walk short distances without aids (Supplementary Table 1). Over half of the respondents (86; 52.8%) reported that ataxia causes permanent problems that restrict their daily activities most of the time, followed by 55 (33.7%) people who experience frequent problems that limit their activities (Supplementary Table 2). Notably, the burden of ataxia on people’s lives has progressed over the years and even decades since their diagnosis (Supplementary Table 3 and supplementary Fig. 1).

**Table 1:** Survey respondent demographics

Respondent type (*n* = 173)	*Patient*	131 (75.7%)
	*On behalf of the patient*	42 (24.3%)
Age distribution (*n* = 173)	*16–29*	22 (12.72%)
	*30–59*	115 (66.5%)
	*60–80*	35 (20.2%)
	*80* +	1 (0.6%)
Gender (*n* = 173)	*Female*	93 (53.8%)
	*Male*	80 (46.2%)
Diagnosis (*n* = 159)	*FA*	56 (35.2%)
	*Inherited CA*	42 (26.4%)
	*Idiopathic CA*	19 (12%)
	*Other types*	27 (17%)
	*Not known*	15 (9.4%)
Attendance to SAC (*n* = 139)	*Non-SAC*	27 (19.4%)
	*SAC*	82 (59%)
	*Used to SAC*	30 (21.6%)

*CA* cerebellar ataxia, *FA* Friedreich’s ataxia. Other types include cerebrotendineus xanthomatosis, ataxia with oculomotor apraxia type 1, Gordon Holmes cerebellar ataxia, POLR3-related spastic ataxia, vitamin E deficiency, cerebellar ataxia neuropathy and vestibular areflexia syndrome, cerebellar ataxia, fragile X-associated tremor/ataxia syndrome, spastic ataxia 9, multiple system atrophy, and multiple system atrophy of cerebellar type C

### Reaching a diagnosis

Most participants (*N* = 120; 76%) reported having a confirmed diagnosis that involved genetic or other tests (Supplementary Table 4). These diagnoses were given for 55.7% patients in a SAC, 32.9% in a non-SAC neurology clinic, and 5.7% in other healthcare services (Supplementary Table 5). There was a greater proportion of definite diagnoses, i.e., FA, specific inherited CAs and other identified types, in the SAC group than in the non-SAC group (Supplementary Fig. 2). Among the participants with a definite diagnosis, 50.6% in the SAC group, 57.2% in the used to SAC group, and 48.15% in the non-SAC group reached a confirmed diagnosis within one year after seeing a neurologist (Supplementary Fig. 3).

### Patient pathway

#### Referral to a neurologist

We asked participants how long they waited to be referred to a neurologist after seeking medical advice for their symptoms: 44% (*N* = 61) were referred within six months (Supplementary Table 6). For the first referral to see a neurologist, 56.2% (*N* = 77) of the participants went directly to a SAC (Supplementary Table 7).

#### Access to the SAC

The participants reported different ways of accessing a SAC: 52.6% (*N* = 51) were referred by a neurologist from a non-specialist clinic, 17.5% (*N* = 15) by a family doctor, and 14.4% (*N* = 14) by other HCPs (Supplementary Table 8). Other ways to access SACs include engaging through charity channels and self-referral. Among those who accessed a SAC (participants in the SAC and used to SAC groups), 51% went to a non-specialist clinic before going to a SAC (Supplementary Table 9). For those who never attended a SAC, the top reasons were that a referral was not offered (26.3%; *N* = 20) and that the centres were too far away (14.7%; *N* = 11) (Table 2). Travel as a barrier to accessing a SAC was also listed as the top reason why people stopped going to a SAC (26.7%; *N* = 12) (Supplementary Table 10). Other reasons why people stopped going were: the local care they received was equivalent (17.8%; *N* = 8), the service was not useful (17.8%; *N* = 8) and other reasons (20%; *N* = 9), which again included the mention of living far from a SAC being an issue.

**Table 2:** Reasons why people never visited a SAC

Reasons	Responses *N* (%)
Current level of care is sufficient	6 (8)
I asked to be referred to a SAC but was refused by my doctor	0 (0)
SACs are too far away for me to travel to	11 (14.7)
Did not wish to be referred	6 (8)
A referral to SAC was not offered to me	20 (26.7)
Other [please specify in the text box]	4 (5.3)
Not applicable	22 (29.3)
Unsure	6 (8)
Do not wish to answer	0 (0)
Total N respondents	75 (100)

Comments: The neurologist at Besta Institute/Milan collaborates with the specialist ataxia centre but he didn't consider it useful to send the patient to his colleagues, Covid arrived immediately after the diagnosis, too far

#### Access to an MDT

A significant proportion of participants were seen by an MDT for their ataxia (*N* = 54; 45%; Supplementary Table 11A); among them, 65.6% (*N* = 38) found visiting the clinic effective (Supplementary Table 11C). Most of the referrals to an MDT clinic were performed by a neurologist at the local hospital (39.35%; *N* = 24) or by a neurologist at an SAC (39.35%; *N* = 24) (Supplementary Table 11B).

#### Patient experience and satisfaction

Among participants who attended both SAC and non-SAC services, 48.9% (*N* = 46) reported that the care received at a SAC was better (Supplementary Table 12). When comparing their experiences between SAC and non-SAC services, SACs did better in understanding the condition, in helping people cope with their condition, in providing practical advice on how to live better with ataxia, in providing medical advice on the management of ataxia symptoms, in coordinating referrals to other HCPs, in offering opportunities to take part in research, and in communicating with social care professionals (Table 3). SAC services were not better at helping people obtain financial support.

**Table 3:** Participants’ feedback on the comparison of the SAC and non-SAC services

Group *N* (%)	SAC			Used to SAC			Total		
Feedback	Positive	Neutral	Negative	Positive	Neutral	Negative	Positive	Neutral	Negative
Good understanding of your condition	64 (86.5)	10 (13.5)	0 (0)	16 (64)	9 (36)	0 (0)	80 (64)	19 (36)	0 (0)
Total	74 (100)			25 (100)			99 (100)		
Makes you able to cope better with your condition	54 (72)	21 (28)	0 (0)	12 (48)	12 (48)	1 (4)	66 (66)	33 (33)	1 (1)
Total	75 (100)			25 (100)			100 (100)		
Gives practical advice about how to live better with ataxia	57 (78.1)	16 (21.9)	0 (0)	13 (54.15)	10 (46.65)	1 (4.2)	70 (72.2)	26 (26.8)	1 (1)
Total	73 (100)			24 (100)			97 (100)		
Provides medical advice on the management of your symptoms	55 (73.4)	17 (23.6)	0 (0)	13 (54.2)	10 (42.7)	1 (4.2)	68 (70.8)	27 (28.1)	1 (1)
total	72 (100)			24 (100)			96 (100)		
Coordination of referrals to specialists	47 (66.2)	21 (29.6)	3 (4.2)	9 (39.1)	13 (56.5)	1 (4.4)	56 (59.6)	34 (36.2)	4 (4.3)
Total	71 (100)			23 (100)			94 (100)		
Offers to participate in research	60 (85.7)	8 (11.4)	2 (2.9)	9 (45)	11 (55)	0 (0)	69 (76.7)	19 (21.1)	2 (2.2)
Total	70 (100)			20 (100)			90 (100)		
Liaising with social workers	38 (53.5)	30 (42.3)	3 (4.2)	12 (60)	7 (35)	1 (5)	50 (54.9)	37 (40.7)	4 (4.4)
Total	71 (100)			20 (100)			91 (100)		
Help with benefits	26 (38.2)	35 (51.5)	7 (10.3)	8 (40)	11 (55)	1 (5)	34 (38.6)	46 (52.3)	8 (9.1)
Total	68 (100)			20 (100)			88 (100)		

We asked all the participants to rate their experience of primary care services, secondary care, including non-specialist neurology clinics, and SAC services according to two different statements: (1) the HCPs understood how to manage their condition, and (2) the HCPs knew about treatments available for ataxia. The results are summarised in Supplementary Tables 13a-f. Overall, the feedback from the SAC group tends to be more positive across the services, particularly for secondary care, compared to people in the non-SAC group. The participants’ experiences with SAC services were better than other services: 71 (75.5%) responded positively to statement 1 and 69 (75%) responded positively to statement 2.

### Unmet needs

The needs that participants expressed to improve the care they received for their ataxia are summarised in Supplementary Table 14. People would like some improvement in the information shared about their condition and treatments available. They wish to feel more in control by receiving more advice on how to cope better and gain better access to holistic treatments. Managing the condition at home by accessing the same level of care locally, if people are no longer able to visit a SAC, was also valuable.

The needs expressed by participants from both the SAC group and the non-SAC group were quite similar (Supplementary Table 15), with a trend toward a greater need for an earlier diagnosis in the SAC group together with more advice on how to live with ataxia. On the other hand, people in the non-SAC group reported more needs related to managing their symptoms better.

### Resource use and costs

The most common contacts for SAC and non-SAC patients were physiotherapy visits and SLT (speech and language therapy) visits (Table 4). The differences in the number of contacts for the different types of health services between the SAC and non-SAC groups were mostly non-significant. The exception was the visits to the neurologist (non-SAC services) with greater mean contacts for patients in the non-SAC group than for those in the SAC group (unadjusted analysis: *P* < 0.01; adjusted analysis: *P* = 0.02). The mean total cost per patient over a one-year period was €1952 for non-SAC patients and €1666 for SAC patients (*P* = 0.79; adjusted analyses).

**Table 4:** Health care contacts over a one-year period for non-SAC and SAC patients

	Patients who reported never attending a SAC				Patients who reported attending a SAC currently						
Health care contacts	*N*	Mean	Std. Dev	Median	*N*	Mean	Std. Dev	Median	Unit cost	*P* value†	*P* value‡
Specialist centre visits	22	0	0	0	74	1.4	0.7	1	39	< 0.01	< 0.01
Family doctor visits	19	1.3	2.6	0	54	0.7	1.3	0	28	0.20	0.39
Neurologist visits	20	2.7	5.0	1.5	55	0.4	0.7	0	29	< 0.01	0.02
Inpatient stays	19	0.3	0.6	0	57	0.3	0.6	0	5575	0.91	0.51
Emergency room visits	18	0.3	0.8	0	57	0.1	0.4	0	29	0.30	0.17
Physiotherapy visits	21	18.9	19.6	10	63	14.4	19.8	2	25	0.37	0.39
Speech and language therapy visits	19	7.8	11.6	1	61	6.4	12.7	1	25	0.67	0.78
Occupational health therapy visits	18	1.5	2.6	0	50	1.3	4.0	0	25	0.88	0.95
Other consultant specialist visits	19	2.3	4.0	0	59	2.1	6.5	1	29	0.91	0.34
Total cost	15	1952	3259	1145	37	1666	3300	321		0.78	0.79

^†^ Test for significant differences in mean values between the non-SAC and SAC groups (unadjusted)

^‡^ Test for significant differences in mean values between the non-SAC and SAC groups (adjusted for age, sex, number of symptoms and number of comorbidities)

*SAC* specialist ataxia centre, *N* number of participants who responded to that question

When we stratified our analyses by the presence of comorbidities, these trends remained the same for those with and without comorbidities (Supplementary Table 16). The mean total cost per patient over a one-year period was for no comorbidities: €3023 for non-SAC patients and €1981 for SAC patients (*P* = 0.79); for patients with comorbidities, €1391 for the non-SAC group and €565 for the SAC group (*P* = 0.56). For non-SAC patients, there were no differences in resource use or costs between those with and without comorbidities; the same was true for patients in the SAC group except for inpatient stays (*P* = 0.04). We surmise that the lower mean costs among those with comorbidities could be due to the small numbers of observations in these groups.

We also examined differences in costs according to the number of symptoms related to ataxia. The numbers in the “No symptoms” and “5–8 symptoms” groups were small, so caution is needed when examining these results, which are summarised in Supplementary Table 17.

#### Patient travel

For the SAC group, the modal travel time was less than 1 h (Supplementary Table 18), although a small proportion of patients travelled for more than 4 h. This finding is in line with the reported living locations of participants in the regions where SACs are located (Supplementary Table 19). The most common mode of transport to a SAC was via a car (64% of patients). For both non-SAC and SAC patients, the modal travel time to see the neurologist at the non-specialist clinic was less than 1 h (40% and 67%, respectively), and most travelled by car (69% and 86%, respectively).

## Discussion

This study examined patient reported care pathways, access to specialist services, and associated healthcare costs for people living with ataxia in Italy, with a focus on the role of Specialist Ataxia Centres.

### Patient pathway

When ataxia symptoms occur, it is important that people see a neurologist [26]. This study revealed a similar proportion of participants who saw a neurologist within 6 months of seeking medical advice as our survey did in the UK (46.4%) and Germany (48.3%) [22, 23]. In Italy, the referral rate to SAC as the first referral to see a neurologist is much higher (56.2%) than in Germany (18%). In both countries, we note different ways to access a SAC, including HCP referrals and self-referrals, which differ from those in the UK, where accessing a SAC is strictly through a referral by an HCP [22]. This might reflect a role and use of SACs in Italy comparable to those in Germany and different from those in the UK. Many centres in Italy are widely distributed across the country. Patients tend to visit such centres once a year or every two years (personal communication with focus groups). For ongoing care, the family doctor oversees the management of the symptoms and refers to local care specialists. In contrast, in the UK, SACs play a central role in referring to other specialists.

Across the three countries we studied, it has been challenging to determine the role of SACs in the diagnosis of ataxia. In Italy, there are more people with a specific diagnosis in the SAC group than in the non-SAC group. However, we did not collect evidence to ascertain whether SACs play a role in facilitating the diagnostic process. And in SAC services patients expressed their need to achieve a definite diagnosis earlier.

In Italy, the proportion of people who access an MDT clinic is the highest of the three countries [22, 23], with referrals done at similar rate by SAC and non-SAC neurologists. Like in other countries, people were satisfied with the care they received there. In Italy, MDT clinics are part of the SAC service, without any specific funding scheme for these clinics. The professionals running such clinics are usually based at the hospital where the SAC is (communication from the focus group).

### Patient satisfaction

In this cohort, 49% of people who went to non-specialist clinics before going to the SAC said that the care received in the SAC was better. This feedback is proportionally less positive than that in the UK and Germany [22, 23]. However, looking at the specific feedback about the various aspects of SAC service, people reported that they delivered better service in most aspects (coordinating referrals, offering opportunities to take part in research, communicating with social care professionals). As expected, patient satisfaction increased with the level of disease specific expertise, from primary to tertiary care settings.

### Resource use and costs

Our findings from a partial and exploratory health economic evaluation suggest that SAC patients are less likely to see a neurologist in a non-specialist clinic than non-SAC patients are. There were no differences in the total costs between the non-SAC and SAC groups, suggesting that the cost of the additional visits to the SAC in the SAC group was offset by the cost savings from fewer neurologist visits at non-specialist clinics.

The time to travel to SAC or non-SAC services was comparable for the participants from both the SAC and non-SAC groups, with 40% under 1 h. However, one of the reasons why 26.7% of the participants stopped attending a SAC was travel. Compared with Germany and the UK, travel was a barrier despite the greater number of centres across Italy [22, 23]. This suggests that the number of centres is not enough to facilitate patients’ adherence to attend SAC. A combination of telemedicine and in-person attendance at SAC services could be a solution to maintain ataxia patients’ care under SAC.

### Study limitations

The participants were recruited via AISA channels and by clinicians in SACs. The recruitment through clinicians to maximise the number of participants may have led to a bias in the way these people answered the questionnaires compared to the people recruited independently of the SACs. The people recruited in SACs received the survey form and instructions from HCPs working at SACs on how to complete the survey. Following that step, the patients completed the form themselves. The survey was not designed to capture the full economic burden of ataxia, but rather patient‑reported healthcare resource use. The costs reported are defined as direct healthcare costs only, reflecting services reimbursed by the Italian National Health Service (Servizio Sanitario Nazionale) and do not include direct non‑medical (e.g. mobility aids, home adaptations) or indirect costs (e.g. employment loss). Another limitation of this study is the small number of respondents to the questions on healthcare service use, which made statistical analyses of differences between groups difficult to interpret, particularly when we investigated differences in participants stratified by the presence of comorbidities and the number of symptoms associated with ataxia. Due to anonymisation of the survey responses, duplicate entries cannot be entirely excluded although the survey platform restricted multiple entries from the same computer or browser. The survey took place shortly after prolonged COVID 19 restrictions, which may have influenced responses, particularly regarding travel and access to specialist centres.

### Recommendations

We collected similar feedback in this cohort compared with that in Germany and the UK regarding people’s needs for better care: improving the information delivered about ataxias, better treatment, feeling more in control with more advice on how to cope better, better access to therapies, and access to effective home care and local care if people can no longer attend the SAC. Further discussion with members of the focus group led to recommendations at various stages of the patient’s journey. First, it is crucial to develop better communication methods between SACs to share common diagnostic and treatment protocols. Thus, an optimised approach for care would require better coordination between SACs and primary and secondary care providers to better organise the monitoring of ataxia patients and continuity of care locally. With a multidisciplinary approach involving patients, family members and doctors, the care delivered would have a greater impact in terms of better symptom management and improved quality of life.

The findings of this study highlight an important distinction between theoretical entitlement to care and effective access to specialist services within a universalistic National Health Service (NHS). While the Italian health system, following the model of the NHS, provides universal coverage and a relatively wide distribution of Specialist Ataxia Centres, universal access does not necessarily translate into equitable or timely access to specialised care for people living with ataxia. Geographic factors, travel burden, and the organisation of referral pathways continue to influence patients’ ability to benefit from specialist services [27]. Our results show that the availability of SACs alone is insufficient if care is not effectively integrated with primary and secondary services and adapted to patients’ functional limitations. These observations suggest that the added value of a universal NHS in the context of rare neurological diseases lies not only in coverage, but in the coordination, continuity, and flexibility of care delivery across settings, including the integration of local services and remote consultations to support long‑term management.

## Conclusion

This study explores the patient pathway and costs associated with the treatment and care received by ataxia patients attending SACs versus those receiving non-specialist clinics in Italy. Our cost analysis combined with results on the care pathway show that the management of ataxia at a SAC can lead to better care and improved patient satisfaction without increasing the cost of treatment. Italy is well provided with several SACs across the country, yet travelling appeared to be a barrier for the attendance and adherence of patients to SAC services. Making expertise accessible locally and supporting the implementation of care plans where people live by providing a combination of face-to-face and telemedicine could improve patients’ attendance at SACs and ultimately support them in better managing their ataxia. Further research would be useful to explore the impact of being treated at a SAC on health-related quality of life and other costs associated with living with ataxias, e.g., out-of-pocket expenses and time off work.

## Supplementary Information

Below is the link to the electronic supplementary material.

Supplementary file1 (DOCX 36.7 KB)

Supplementary file2 (DOCX 5053 KB)

## Data Availability

The data underlying this article will be shared on reasonable request to the corresponding author.
