# Supplementary material for: Ataxia patient care pathway and associated healthcare costs in Italy: a cross-sectional survey
Source: Neurol Sci. 2026 Jul 16;47(8):638. doi: 10.1007/s10072-026-09150-w (PMC13375654; doi:10.1007/s10072-026-09150-w)

Supplementary Table 1: Level of mobility of the participants

| **Answer choices** | **Responses N (%)** |
| --- | --- |
| No functional restrictions | 1 (0.6) |
| Mild impairment, I can walk and run without restriction | 12 (6.9) |
| Moderate impairment, I cannot run, I can walk a limited distance without aids | 43 (24.7) |
| I need a stick to walk | 17 (9.8) |
| In the house I need two sticks / a rollator, outside I need a wheelchair | 28 (16.1) |
| I can’t walk and need a wheelchair, but otherwise I don’t need help | 14 (8.0) |
| I can’t walk and need a wheelchair, and I am dependent on help | 57 (32.7) |
| I am bedridden | 2 (1.2) |
| **Total** | 174 (100) |

Supplementary Table 2: Burden of ataxia on people’s lives

| **Answer choices** | **Responses N (%)** |
| --- | --- |
| My ataxia doesn’t limit me | 4 (2.5) |
| My ataxia causes problems every now and then | 18 (11) |
| My ataxia causes frequent problems and limits my activities | 55 (33.7) |
| My ataxia causes permanent problems and restricts me most of the time | 86 (52.8) |
| I do not know | 0 (0) |
| **Total** | 163 (100) |

Supplementary Table 3: Level of severity at the beginning of the pathway, when the first diagnosis was given


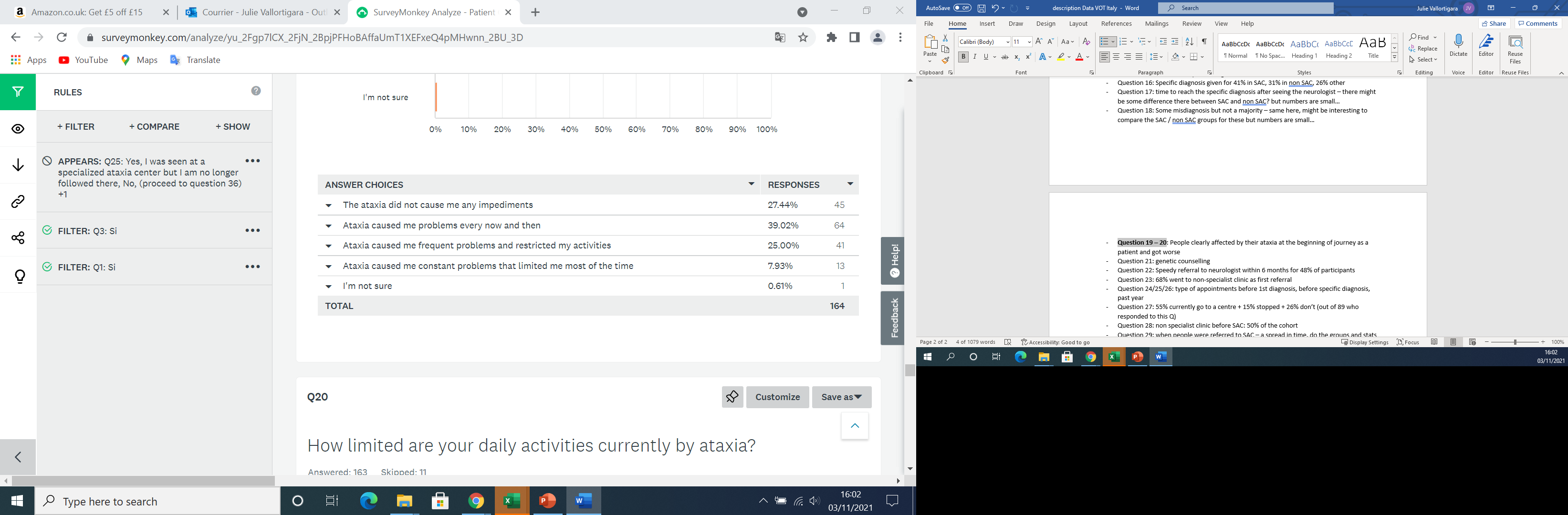


Supplementary Figure 1: Time spent living with ataxia since first diagnosis was given by a Healthcare professional

1: 1-5 years; 2: 6-10 years; 3: 11-15 years; 4: 16-20 years; 5: 21-25 years; 6: 26-30 years; 7: 31-35 years; 8: 36-40 years; 9: 41-45 years; 10: 46-50 years.

Supplementary Table 4: Proportion of participants who received a confirmed diagnosis with genetic or other tests


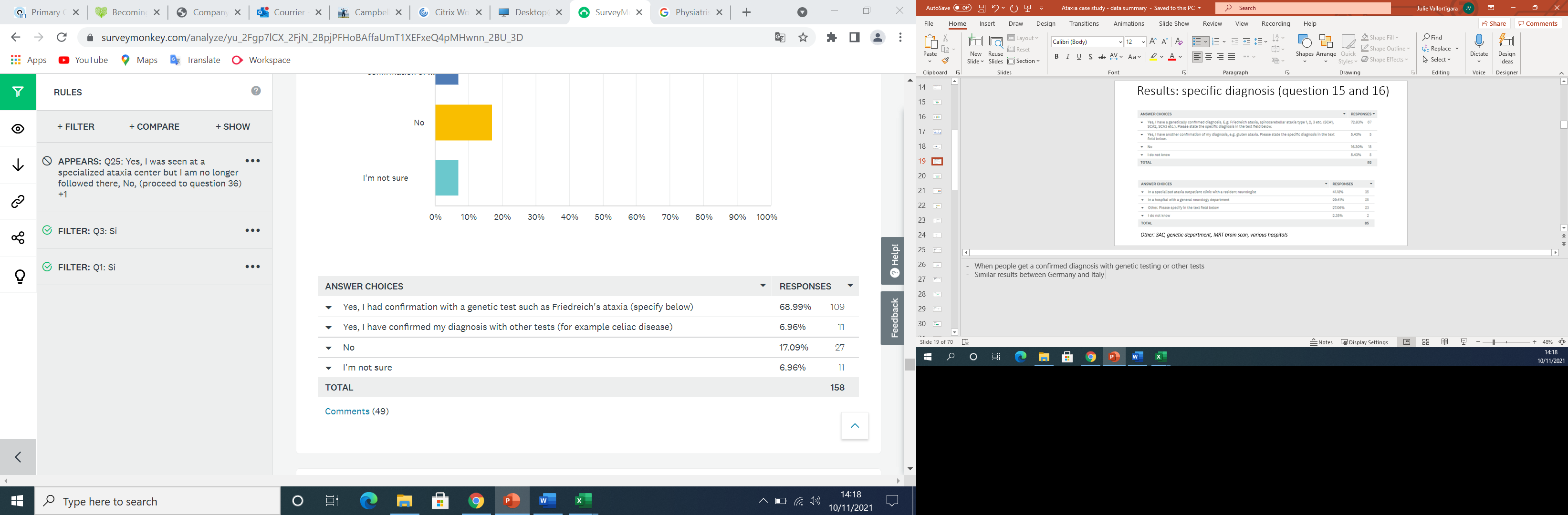


Supplementary Table 5: Place where participants received their confirmed diagnosis of ataxia


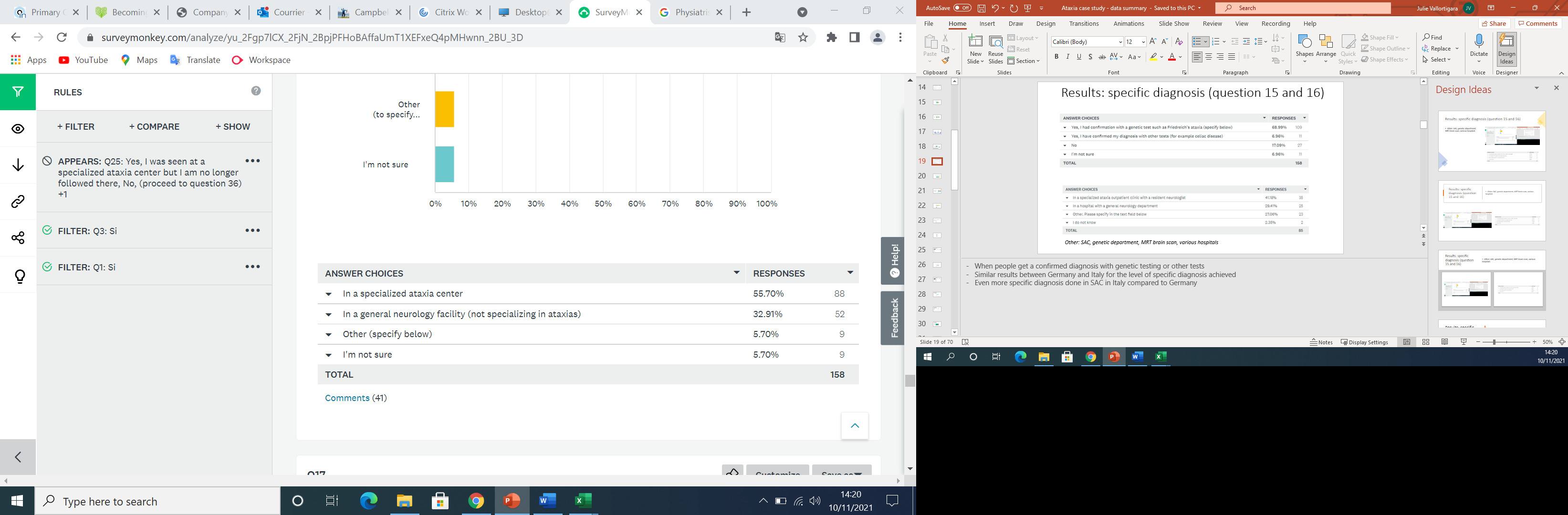


Other: Besta institute Milan, various polyclinics and hospitals, General children Neuropsychiatric clinic and children Hospitals, genetics department

Supplementary Figure 2: Confirmed diagnosis by SAC attendance


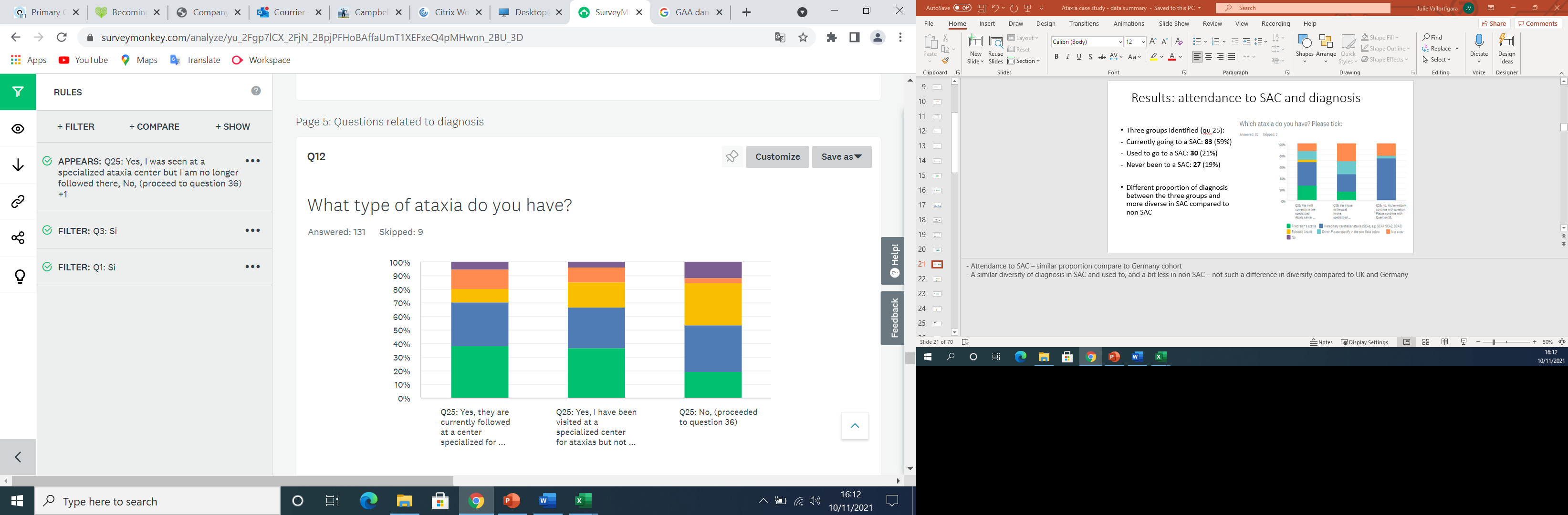


Figure 2 bis: Time between neurologist visit and specific diagnosis given by SAC attendance


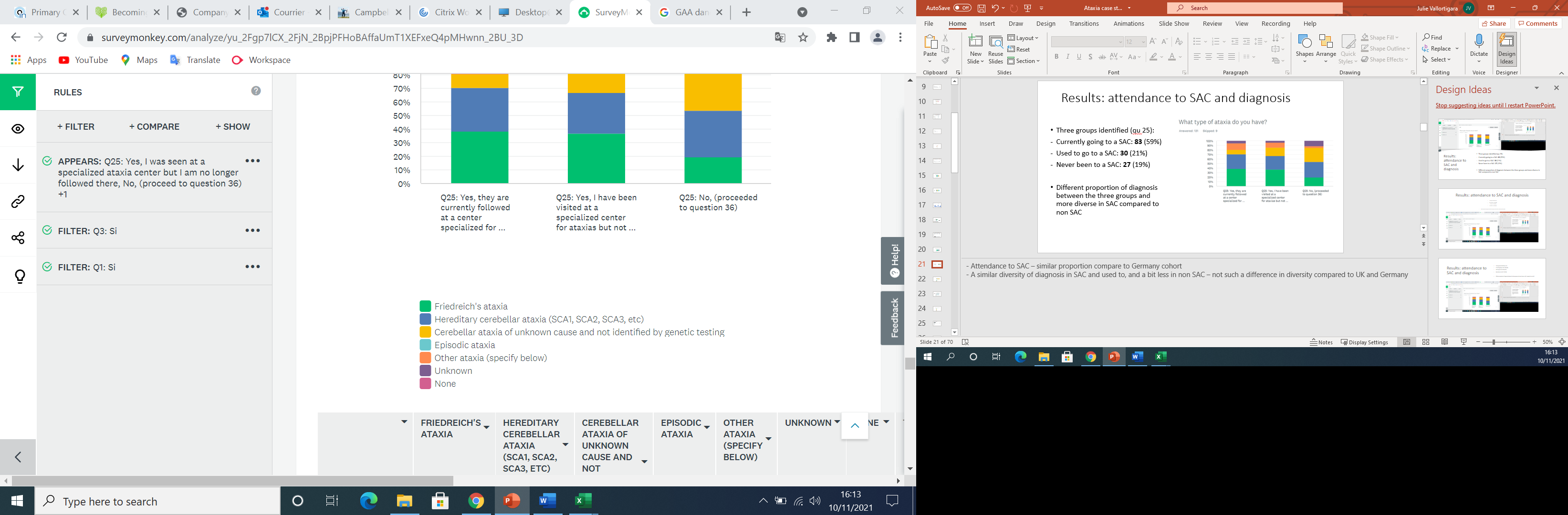


Supplementary Figure 3: Time between neurologist visit and specific diagnosis given by SAC attendance


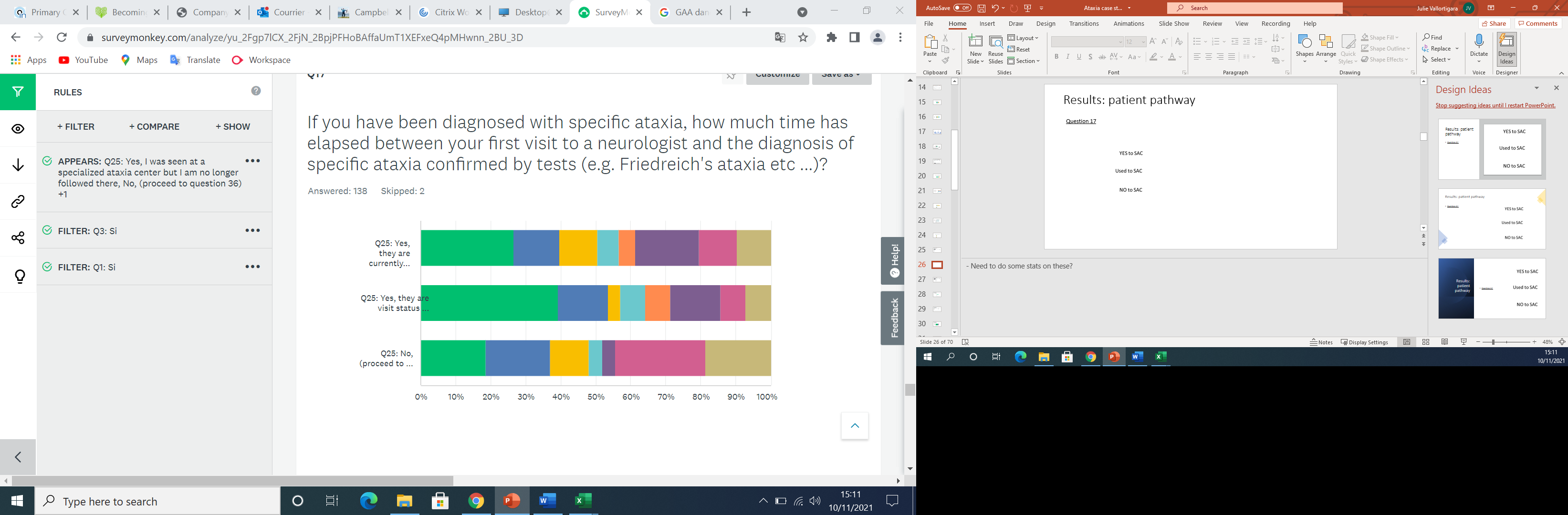


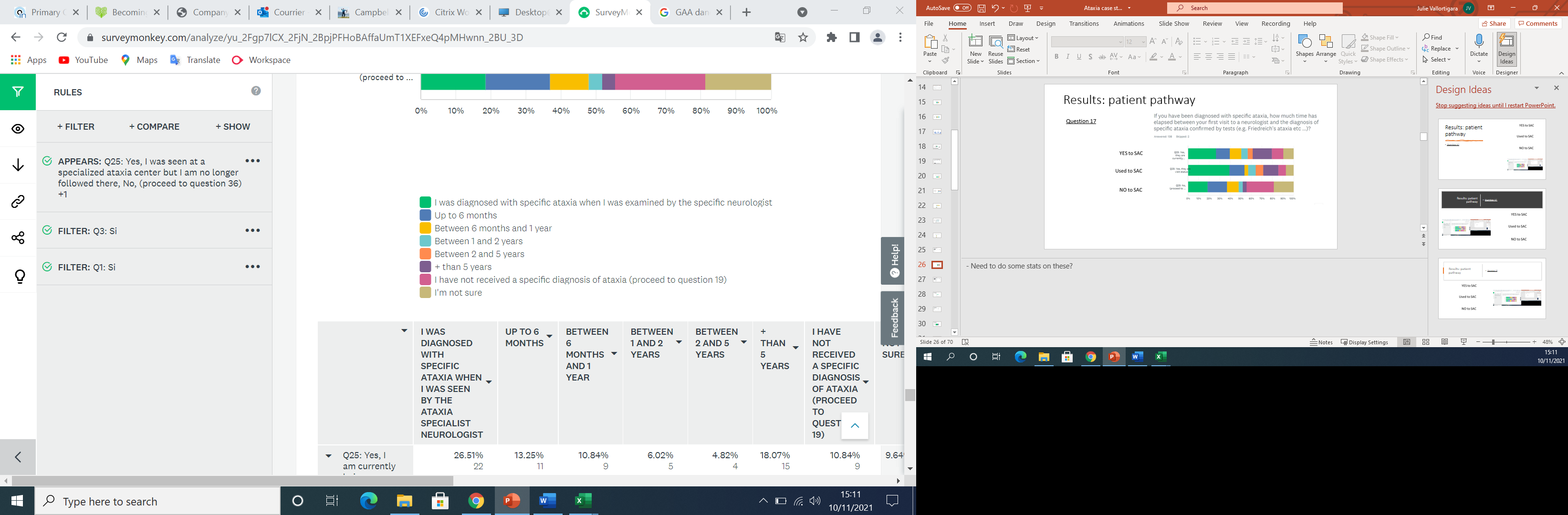


Supplementary Table 6: Time spent between first medical advice on ataxia symptoms and referral to see a neurologist


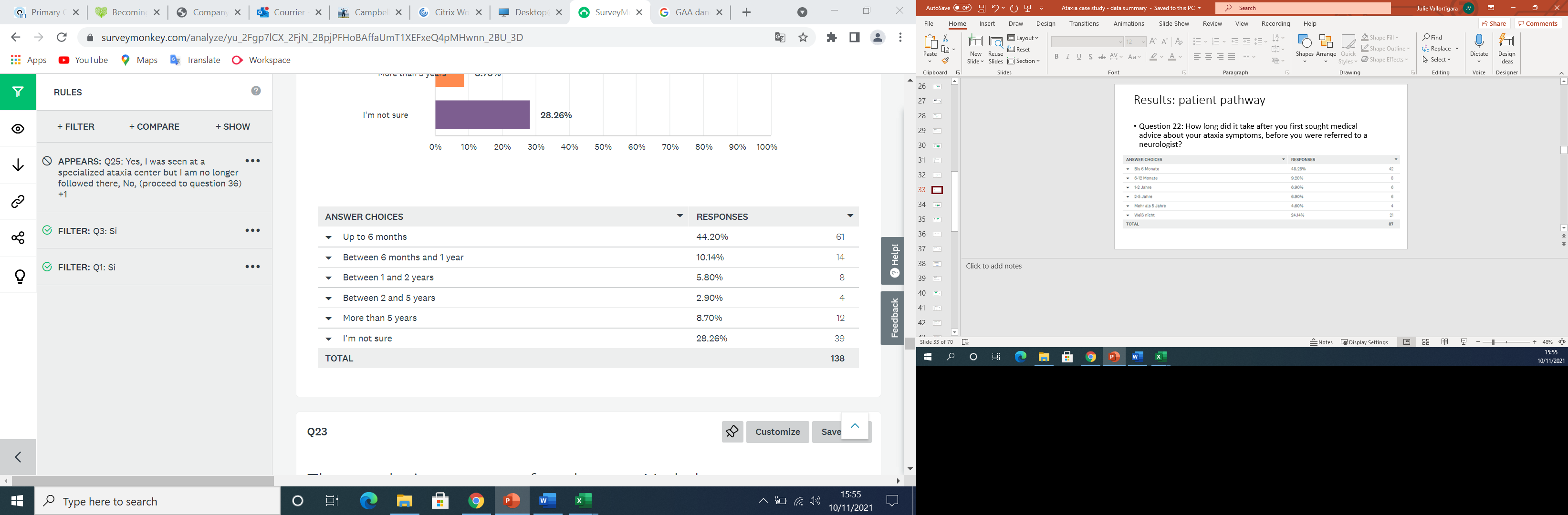


Supplementary Table 7: Where was the neurologist participants saw for their first referral


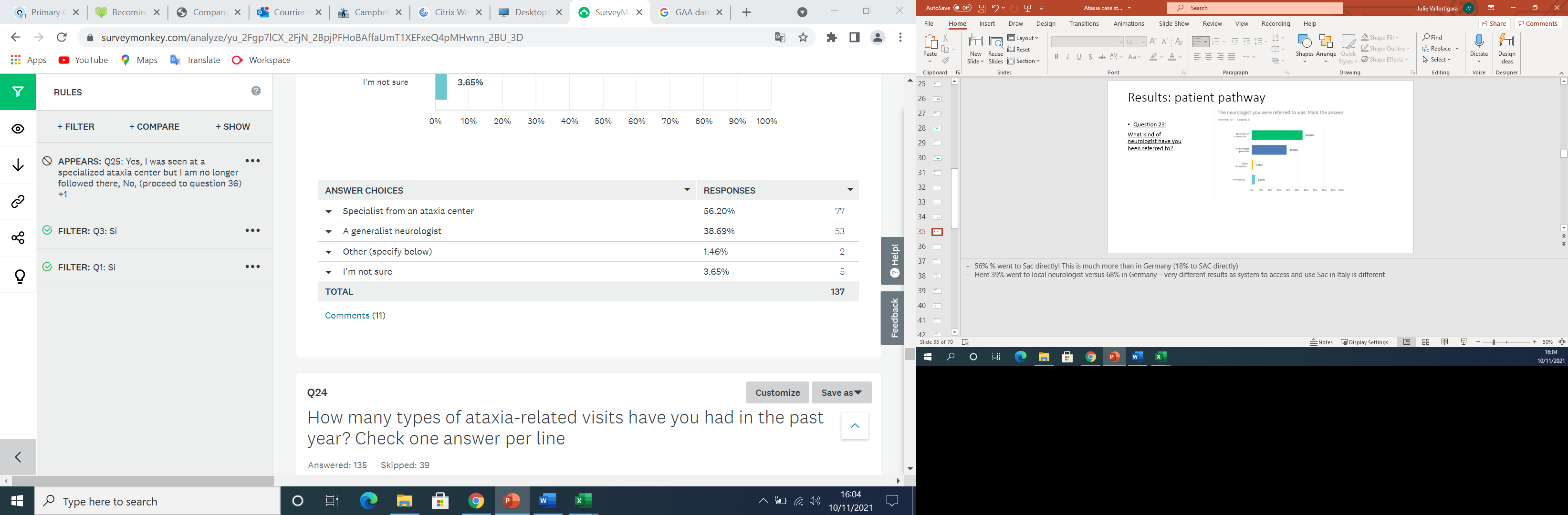


Supplementary Table 8: Who referred the participants to a SAC

| **Answer choices** | **Responses N (%)** |
| --- | --- |
| GP | 15 (17.5%) |
| Neurologist | 51 (52.6%) |
| Other HCP | 14 (14.4%) |
| Unsure | 17 (17.5%) |
| **Total** | **97 (100%)** |

Comments on ways to access a SAC: on my own, a cousin perhaps, voluntarily for RG283 testing, psychiatrist colleague, GP first and then Hospital neurologist, Orthopaedist, a friend, telethon operator, Physiatrist, Neurologist and I'll go on July, Neurologist /private clinic, On 2017 I changed Neurologist and I went on my own, I requested for an appointment c/o Besta Institute/Milan, While speaking with physiotherapist, Association, We saw AISA tv presentation linked to the event " la Vela per la Vita" speaking at ataxia and Besta Institute /Milan.

Supplementary Table 9: participants who went to a non-specialist neurology clinic before going to a SAC


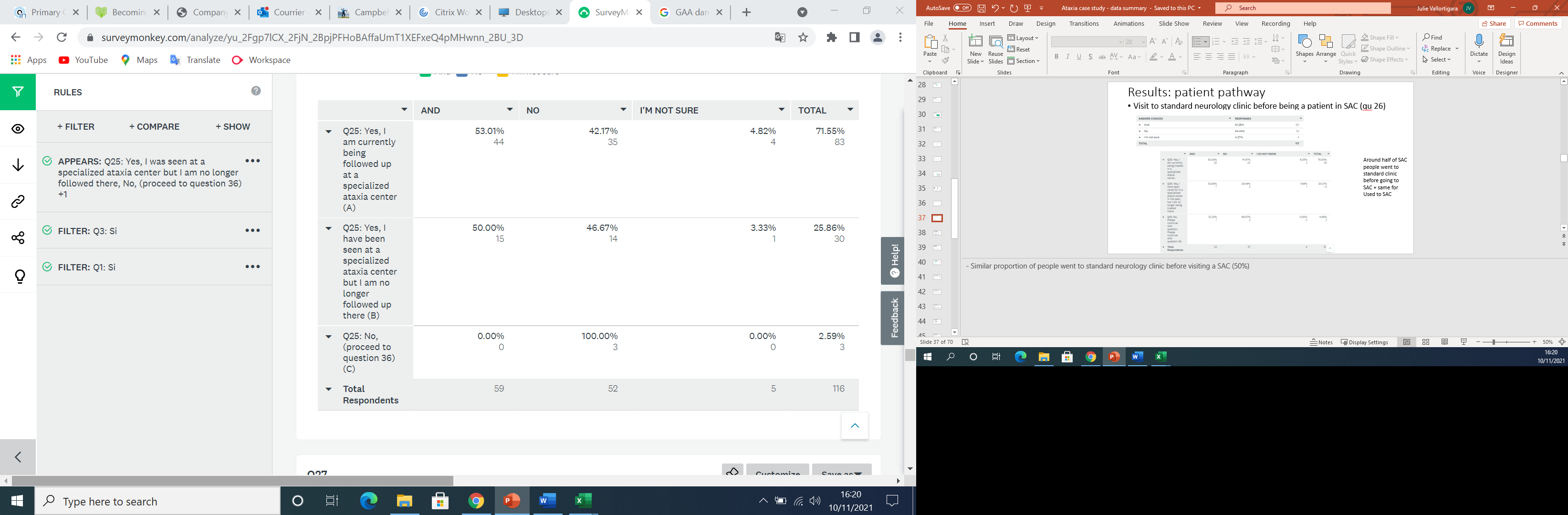


Supplementary Table 10: Reasons why people stopped going to a SAC


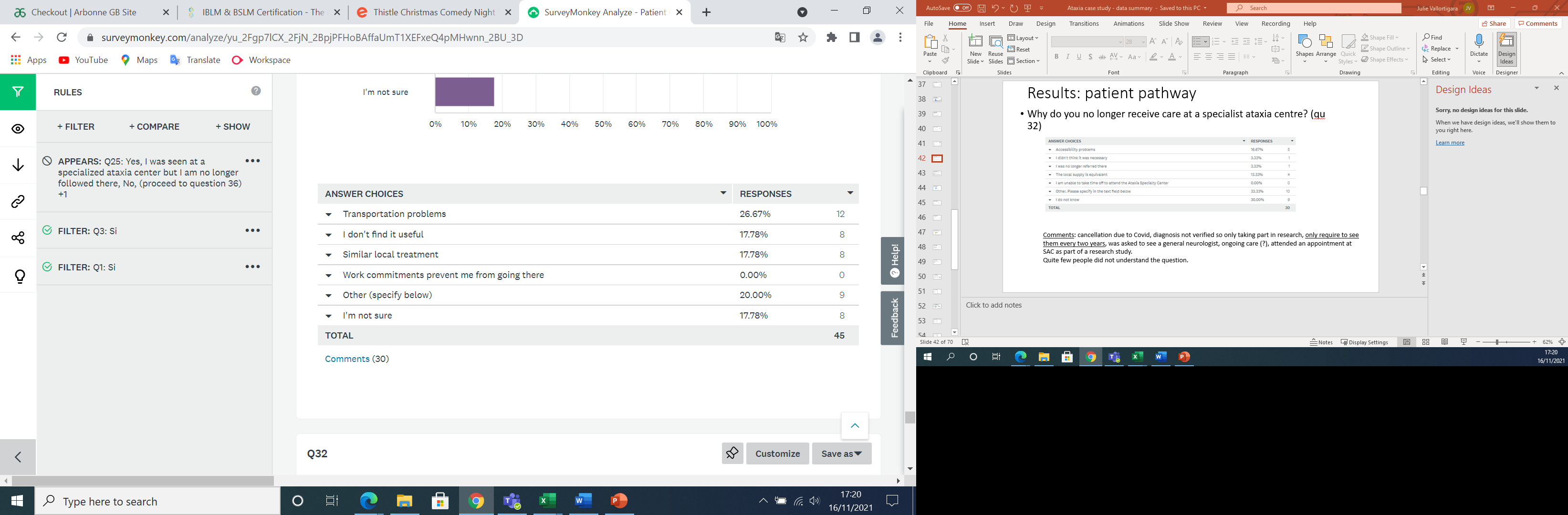


Comments: I am disappointed with the answers from the specialists, Latina is far from Rome, C/O Besta Institute/Milan they told me that at Ferrara hospital they can take care of me. At Besta they couldn't do nothing more, far from the centre- Transport problems, I attend a non-ataxia specialist daily association but I am well followed up and they well understand my condition, I am still occasionally followed up, During the first years they seem to help then nothing, I hope they could do more they simply gave me a diagnosis suggesting me to refer to a neurologist I trust, I lost my contacts and not yet found an ataxia specialist who can follow me up in Puglia or near; stopped due to Covid.

Supplementary Table 11: Visit to a Multidisciplinary clinic

A: attendance

| **Answer choices N (%)** | **YES** | **NO** | **Unsure** | **Total** |
| --- | --- | --- | --- | --- |
| SAC | 34 (50) | 29 (42.6) | 5 (7.4) | 68 (100) |
| Used to SAC | 14 (51.9) | 11 (40.7) | 2 (7.4) | 27 (47.5) |
| Non-SAC | 6 (25) | 17 (68) | 2 (8) | 25 (7.5) |
| **Total** | **54 (45)** | **57 (47.5)** | **9 (7.5)** | **120 (100)** |

B: referral

| **Answer choices** | **Responses N (%)** |
| --- | --- |
| Neurologist at SAC | 24 (39.35) |
| Neurologist (non-specialist clinic) | 24 (39.35) |
| Other | 13 (21.3) |
| **Total** | **61 (100)** |

Comments: Physiatrist, AISA, Ataxia Centre neurologist, physiotherapist, partner, myself, My own research of the Multidisciplinary team, My wife asked for physiatrist outpatient visit and activated physiotherapy and speech therapy, GP, geneticist, Aisa Lazio, friends.

C: Feedback of participants on the effectiveness of the multidisciplinary team care they received

| Feedback | Positive N (%) | Neutral  N (%) | Negative N (%) |
| --- | --- | --- | --- |
| Number of respondents | 38 (65.6) | 14 (24.1) | 6 (10.3) |
| Total respondents | 58 (100) | | |

Supplementary Table 12: Was the care delivered at a SAC an improvement compared to the care in standard neurology clinic

| Answer choices | responses N (%) |
| --- | --- |
| Yes | 46 (48.9%) |
| No | 39 (41.5%) |
| Unsure | 9 (9.6%) |
| Total | 94 (100%) |

Supplementary Table 13a: Participants agreement rate on primary care health professionals (e.g. GP, physiotherapist, occupational therapist) understood how to manage their ataxia

| Feedback | Positive N (%) | Neutral  N (%) | Negative N (%) | Total  N (%) |
| --- | --- | --- | --- | --- |
| SAC | 31(48.4) | 16 (25) | 17(26.6) | 64 (100) |
| Non-SAC | 10 (41.65) | 4 (16.4) | 10 (41.65) | 24 (100) |
| USED to SAC | 7 (30.4) | 8 (34.8) | 8 (26.6) | 23 (100) |
| Total | 48 (43.3) | 28 (25.2) | 35 (31.5) | 111 (100) |

Supplementary Table 13b: Participants agreement rate on primary care health professionals (e.g. GP, physiotherapist, occupational therapist) understood the treatments available for their ataxia

| Feedback | Positive N (%) | Neutral  N (%) | Negative N (%) | Total  N (%) |
| --- | --- | --- | --- | --- |
| SAC | 25 (42.4) | 14 (23.7) | 20 (33.9) | 59 (100) |
| Non-SAC | 8 (32) | 5 (20) | 12 (48) | 25 (100) |
| USED to SAC | 8 (33.3) | 7 (29.2) | 9 (37.5) | 24 (100) |
| Total | 41 (38) | 26 (24) | 41 (38) | 108 (100) |

Supplementary Table 13c: Participants agreement rate on secondary care health professionals (e.g. neurologist, other consultants at my local hospital) understood how to manage their ataxia

| Feedback | Positive N (%) | Neutral  N (%) | Negative N (%) | Total  N (%) |
| --- | --- | --- | --- | --- |
| SAC | 33 (55.9%) | 18 (30.5%) | 8 (13.6%) | 59 (100) |
| Non-SAC | 7 (30.4%) | 7 (30.4%) | 9 (39.2%) | 23 (100) |
| USED to SAC | 9 (34.6%) | 10 (38.5%) | 7 (26.9%) | 26 (100) |
| Total | 49 (45.4%) | 35 (32.4%) | 24 (22.2%) | 108 (100) |

Supplementary Table 13d: Participants agreement rate on secondary care health professionals (e.g. neurologist, other consultants at my local hospital) understood the treatments available for their ataxia

| Feedback | Positive N (%) | Neutral  N (%) | Negative N (%) | Total  N (%) |
| --- | --- | --- | --- | --- |
| SAC | 33 (55.9) | 17 (28.8) | 9 (15.3) | 59 (100) |
| Non-SAC | 8 (36.8) | 6 (47.4) | 8 (15.8) | 22 (100) |
| USED to SAC | 7 (36.4) | 9 (27.3) | 3 (36.4) | 19 (100) |
| Total | 48 (48) | 32 (32) | 20 (20) | 100 (100) |

Supplementary Table 13e: Participants agreement rate on the specialists at SAC understood how to manage their ataxia

| Feedback | Positive N (%) | Neutral  N (%) | Negative N (%) | Total  N (%) |
| --- | --- | --- | --- | --- |
| SAC | 55 (78.6%) | 12 (17.1%) | 3 (4.3%) | 70 (100) |
| USED to SAC | 16 (66.7) | 5 (20.8) | 3 (12.5) | 24 (100) |
| Total | 71 (75.5) | 17 (18.1) | 6 (6.4) | 94  (100) |

Supplementary Table 13f: Participants agreement rate on specialists at SAC understood the treatments available for their ataxia

| Feedback | Positive N (%) | Neutral  N (%) | Negative N (%) | Total  N (%) |
| --- | --- | --- | --- | --- |
| SAC | 54 (79.4) | 10 (14.7) | 4 (5.9) | 68 (100) |
| USED to SAC | 15 (62.5) | 6 (25) | 3 (12.5) | 24 (100) |
| Total | 69 (75) | 16 (17.4) | 7 (7.6) | 92 (100) |

Supplementary Table 14: Participants feedback on how to improve the care delivered

| **Answer choices** | **Responses N** | **Responses (%)** |
| --- | --- | --- |
| More information about my condition | 48 | 40 |
| More information on available treatments | 65 | 54.2 |
| More help to make me feel in control of my condition (i.e. to cope better) | 64 | 53.3 |
| Knowing my specific diagnosis earlier | 22 | 18.3 |
| Better management of my symptoms | 41 | 34.2 |
| Better practical advice on living with the condition | 56 | 46.7 |
| Better access to therapies (e.g. physiotherapy, speech and language therapy, occupational therapy) | 63 | 52.5 |
| More information on help adapting my home | 20 | 16.7 |
| Help in communicating with my employer | 7 | 5.8 |
| More information about the genetics of my condition/ whether my children or grandchildren are at risk of inheriting ataxia | 20 | 16.7 |
| Continuing the same level of care in my home if I am not longer able to visit an ataxia specialist centre | 41 | 34.2 |
| I am satisfied with my care and do not need improvement | 7 | 5.8 |
| Other please specify | 4 | 3.3 |
| I do not know | 0 | 0 |

Comments on how to improve the care: I need a specific diagnosis, I have been quite well followed up and against ataxia there is little to do, Better practical advice on living with my condition, Overall, I wish to get-in real time - more information about ataxia possible treatments, Make specific trainings to general practitioners, to Md and Healthcare workers in Accident &Emergency mandatory, It is useless, there is no cure currently. I tried patches too!! For the time being I am satisfied with my treatments even if it does need improvement, a cure must be found to improve or block ataxia.

Supplementary Table 15: How do you feel your care could be improved – by SAC attendance


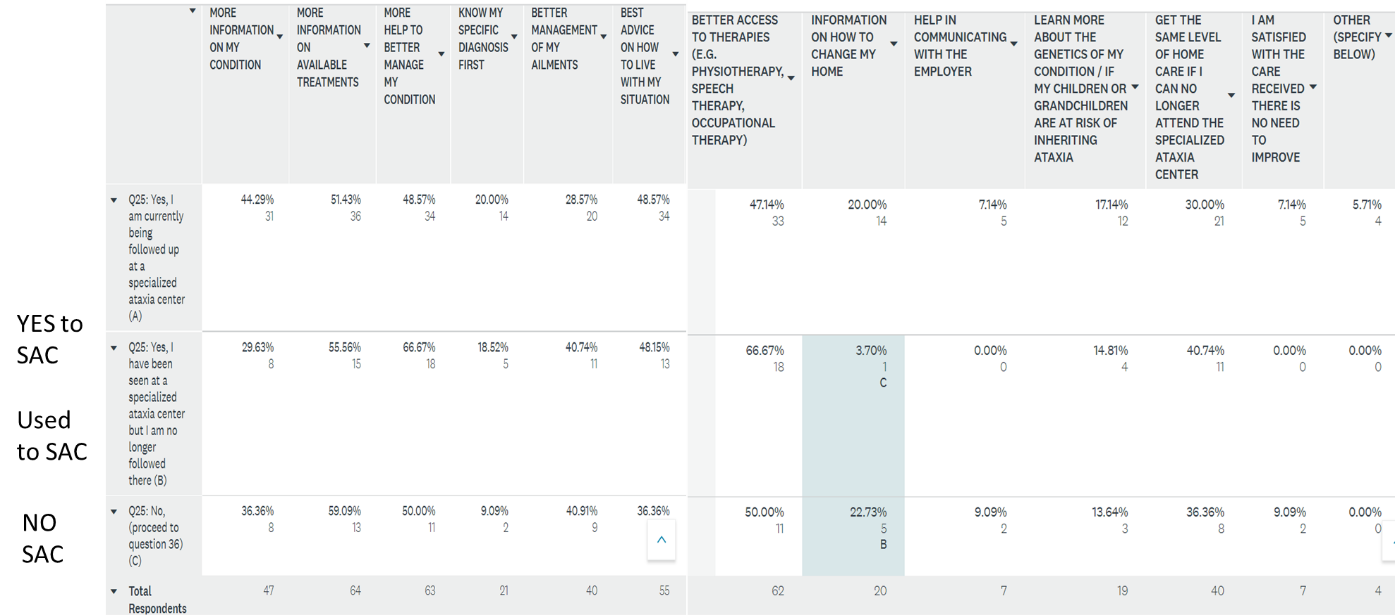


Supplementary Table 16. Health care contacts over a one-year period for non-SAC and SAC patients by presence of comorbidities

|  | **Patients who reported never attending a SAC** | | | | **Patients who reported attending a SAC currently** | | | |  |  |
| --- | --- | --- | --- | --- | --- | --- | --- | --- | --- | --- |
| **Health care contacts** | **N** | **Mean** | **Std. Dev.** | **Median** | **N** | **Mean** | **Std. Dev.** | **Median** | **P-value**† | **P-value**‡ |
| **No comorbidities** |  |  |  |  |  |  |  |  |  |  |
| Specialist centre visits | 13 | 0.0 | 0.0 | 0 | 47 | 1.5 | 0.8 | 1 | <0.01 | <0.01 |
| Family doctor visits | 10 | 0.7 | 0.8 | 0 | 37 | 0.6 | 1.1 | 0 | 0.72 | 0.20 |
| Neurologist visits | 11 | 3.9 | 6.4 | 2 | 37 | 0.4 | 0.7 | 0 | <0.01 | 0.02 |
| Inpatient stays | 9 | 0.3 | 0.7 |  | 39 | 0.3 | 0.6 | 0 | 0.83 | 0.25 |
| Emergency room visits | 9 | 0.3 | 1 | 0 | 36 | 0.1 | 0.3 | 0 | 0.23 | 0.17 |
| Physiotherapy visits | 12 | 19.5 | 18.7 | 10 | 42 | 15.9 | 20.3 | 2 | 0.58 | 0.80 |
| Speech and language therapy visits | 10 | 13.4 | 13.5 | 7 | 42 | 7.2 | 14.3 | 1 | 0.22 | 0.46 |
| Occupational health therapy visits | 9 | 2.5 | 3.3 | 1 | 31 | 0.5 | 1.8 | 0 | 0.02 | 0.13 |
| Other consultant specialist visits | 10 | 3.7 | 5.1 | 1 | 38 | 2.4 | 8.0 | 1 | 0.62 | 0.41 |
| Total cost | 6 | 3023 | 4655 | 1420 | 25 | 1981 | 3832 | 346 | 0.57 | 0.79 |
| **One or more comorbidity** |  |  |  |  |  |  |  |  |  |  |
| Specialist centre visits | 7 | 0 | 0 | 0 | 24 | 1.1 | 0.2 | 1 | <0.01 | <0.01 |
| Family doctor visits | 7 | 1.1 | 2.2 | 0 | 16 | 1.1 | 1.7 | 0 | 0.93 | 0.64 |
| Neurologist visits | 7 | 1.4 | 1.5 | 1 | 16 | 0.3 | 0.8 | 0 | 0.15 | 0.25 |
| Inpatient stays | 8 | 0.1 | 0.3 | 0 | 17 | 0 | 0 | 0 | 0.31 | 0.41 |
| Emergency room visits | 7 | 0.2 | 0.7 | 0 | 19 | 0.1 | 0.5 | 0 | 0.67 | 0.70 |
| Physiotherapy visits | 7 | 22.5 | 23.1 | 12 | 20 | 12.0 | 19.1 | 1.5 | 0.25 | 0.32 |
| Speech and language therapy visits | 7 | 2.0 | 4.4 | 0 | 18 | 4.8 | 8.5 | 1 | 0.41 | 0.50 |
| Occupational health therapy visits | 8 | 0.5 | 1.1 | 0 | 18 | 2.8 | 6.6 | 0 | 0.30 | 0.60 |
| Other consultant specialist visits | 8 | 0.8 | 0.9 | 0 | 20 | 1.3 | 1.5 | 1 | 0.33 | 0.61 |
| Total cost | 8 | 1391 | 1974 | 959 | 11 | 565 | 768 | 118 | 0.22 | 0.56 |
| **P-value**§ |  |  |  |  |  |  |  |  |  |  |
| Family doctor visits | 0.86 |  |  |  | 0.49 |  |  |  |  |  |
| Neurologist visits | 0.97 |  |  |  | 0.95 |  |  |  |  |  |
| Inpatient stays | 0.74 |  |  |  | 0.04 |  |  |  |  |  |
| Emergency room visits | 0.63 |  |  |  | 0.33 |  |  |  |  |  |
| Physiotherapy visits | 0.79 |  |  |  | 0.08 |  |  |  |  |  |
| Speech and language therapy visits | 0.06 |  |  |  | 0.38 |  |  |  |  |  |
| Occupational health therapy visits | 0.40 |  |  |  | 0.05 |  |  |  |  |  |
| Other consultant specialist visits | 0.56 |  |  |  | 0.99 |  |  |  |  |  |
| Total cost | 0.56 |  |  |  | 0.50 |  |  |  |  |  |

† Test for significant differences in mean values between Non-SAC and SAC groups (unadjusted)

‡ Test for significant differences in mean values between Non-SAC and SAC groups (adjusted for age, sex and number of symptoms)

§ Test for significant differences in mean values by comorbidities separately for Non-SAC and SAC groups (adjusted for age, sex and number of symptoms)

A hyphen “-“ indicates that the parameter is not estimable, due to small numbers of observations.

SAC, specialist ataxia centre; N, number of participants who responded to that question.

Supplementary Table 17: Health care contacts over a one-year period for non-SAC and SAC patients by number of symptoms experienced as a result of ataxia

|  | **Patients who reported never attending a SAC** | | | | **Patients who reported attending a SAC currently** | | | |  |  |
| --- | --- | --- | --- | --- | --- | --- | --- | --- | --- | --- |
| **Health care contacts** | **N** | **Mean** | **Std. Dev.** | **Median** | **N** | **Mean** | **Std. Dev.** | **Median** | **P-value**† | **P-value**‡ |
| **No symptoms** |  |  |  |  |  |  |  |  |  |  |
| Specialist centre visits | 3 | 0.0 | - | 0 | 13 | 1.6 | 0.9 | 1 | 0.01 | 0.24 |
| Family doctor visits | 5 | 1.0 | 0.7 | 1 | 8 | 0.5 | 0.7 | 0 | 0.26 | 0.13 |
| Neurologist visits | 4 | 7.0 | 10.8 | 2.5 | 8 | 0.2 | 0.7 | 0 | 0.09 | 0.06 |
| Inpatient stays | 3 | 0.6 | 1.1 | 0 | 11 | 0.3 | 0.6 | 0 | 0.56 | 0.55 |
| Emergency room visits | 3 | 1.0 | 1.7 | 0 | 9 | 0.1 | 0.3 | 0 | 0.14 | 0.73 |
| Physiotherapy visits | 3 | 3.3 | 3.0 | 4 | 12 | 7.5 | 15.1 | 1 | 0.65 | 0.99 |
| Speech and language therapy visits | 3 | 2.3 | 2.0 | 3 | 11 | 7.5 | 16.0 | 0 | 0.60 | 0.93 |
| Occupational health therapy visits | 3 | 1.3 | 2.3 | 0 | 7 | 1.4 | 3.7 | 0 | 0.97 | 0.82 |
| Other consultant specialist visits | 3 | 2.7 | 4.6 | 0 | 11 | 0.8 | 0.8 | 1 | 0.18 | 0.31 |
| Total cost | 3 | 4287 | 7057 | 219 | 6 | 156 | 156 | 93 | 0.17 | 0.66 |
| **1-4 symptoms** |  |  |  |  |  |  |  |  |  |  |
| Specialist centre visits | 12 | 0 | 0 | 0 | 39 | 1.3 | 0.7 | 1 | <0.01 | <0.01 |
| Family doctor visits | 9 | 0.8 | 2.0 | 0 | 28 | 0.5 | 1.2 | 0 | 0.57 | 0.48 |
| Neurologist visits | 10 | 1.2 | 1.0 | 1 | 30 | 0.5 | 0.8 | 0 | 0.04 | 0.04 |
| Inpatient stays | 11 | 0.1 | 0.3 | 0 | 29 | 0.3 | 0.6 | 0 | 0.15 | 0.41 |
| Emergency room visits | 10 | 0.2 | 0.6 | 0 | 30 | 0.1 | 0.4 | 0 | 0.71 | 0.66 |
| Physiotherapy visits | 12 | 23.5 | 21.6 | 20 | 33 | 15.3 | 20.2 | 2 | 0.25 | 0.25 |
| Speech and language therapy visits | 11 | 9.3 | 14.2 | 1 | 31 | 7.3 | 14.2 | 1 | 0.69 | 0.41 |
| Occupational health therapy visits | 10 | 0.5 | 0.9 | 0 | 28 | 1 | 3.5 | 0 | 0.66 | 0.59 |
| Other consultant specialist visits | 10 | 0.6 | 0.8 | 0 | 30 | 2.8 | 9.0 | 1 | 0.46 | 0.55 |
| Total cost | 8 | 935 | 785 | 1210 | 21 | 2450 | 4216 | 321 | 0.33 | 0.69 |
| **5-8 symptoms** |  |  |  |  |  |  |  |  |  |  |
| Specialist centre visits | 2 | 0 | 0 | 0 | 13 | 1.1 | 0.3 | 1 | <0.01 | 0.26 |
| Family doctor visits | 2 | 0 | 0 | 0 | 9 | 1 | 1.7 | 0 | 0.45 | 0.81 |
| Neurologist visits | 2 | 2.5 | 2.1 | 2.5 | 8 | 0.1 | 0.3 | 0 | <0.01 | 0.18 |
| Inpatient stays | 2 | 0 | 0 | 0 | 9 | 0 | 0 | 0 | - | - |
| Emergency room visits | 2 | 0 | 0 | 0 | 10 | 0 | 0 | 0 | - | - |
| Physiotherapy visits | 2 | 11 | 1.4 | 11 | 9 | 30 | 22.8 | 40 | 0.28 | 0.66 |
| Speech and language therapy visits | 2 | 6.5 | 7.7 | 6.5 | 10 | 7.5 | 9.1 | 2.5 | 0.89 | 0.83 |
| Occupational health therapy visits | 2 | 5 | 7.0 | 5 | 10 | 2.6 | 6.3 | 0 | 0.64 | 0.29 |
| Other consultant specialist visits | 2 | 2.5 | 0.7 | 2 | 10 | 2 | 2.2 | 1 | 0.76 | 0.31 |
| Total cost | 2 | 708 | 94 | 708 | 6 | 1265 | 625 | 1415 | 0.28 | 0.56 |
| **P-value**§ |  |  |  |  |  |  |  |  |  |  |
| Family doctor visits | 0.93 |  |  |  | 0.71 |  |  |  |  |  |
| Neurologist visits | 0.17 |  |  |  | 0.30 |  |  |  |  |  |
| Inpatient stays | 0.31 |  |  |  | 0.78 |  |  |  |  |  |
| Emergency room visits | 0.46 |  |  |  | 0.72 |  |  |  |  |  |
| Physiotherapy visits | 0.38 |  |  |  | 0.01 |  |  |  |  |  |
| Speech and language therapy visits | 0.64 |  |  |  | 0.93 |  |  |  |  |  |
| Occupational health therapy visits | 0.01 |  |  |  | 0.39 |  |  |  |  |  |
| Other consultant specialist visits | 0.43 |  |  |  | 0.95 |  |  |  |  |  |
| Total cost | 0.27 |  |  |  | 0.84 |  |  |  |  |  |

† Test for significant differences in mean values between Non-SAC and SAC groups (unadjusted)

‡ Test for significant differences in mean values between Non-SAC and SAC groups (adjusted for age, sex and comorbidities)

§ Test for significant differences in mean values by number of symptoms separately for Non-SAC and SAC groups (adjusted for age, sex and comorbidities)

A hyphen “-“ indicates that the parameter is not estimable, due to small numbers of observations.

SAC, specialist ataxia centre; N, number of participants who responded to that question.

Table 18: Mode of transport and time taken to travel to the SAC and the neurologist based in the general neurology clinic

| **Travel time to visit the Specialist Ataxia Centre (one way) (N=81)** | | | | **Mode of transport mainly used to visit the SAC (N=75)** | | | | |
| --- | --- | --- | --- | --- | --- | --- | --- | --- |
|  | **N** | **%** | |  | | **N** | | **%** |
| Less than 1 hour | 32 | 40 | | Bus | | 2 | | 3 |
| 1 to 2 hours | 29 | 36 | | Car | | 64 | | 82 |
| 2 to 3 hours | 8 | 10 | | Taxi | | 1 | | 1 |
| 3 to 4 hours | 5 | 6 | | Train | | 8 | | 10 |
| More than 4 hours | 6 | 7 | | Other | | 2 | | 3 |
| Unsure | 1 | 1 | | Unsure | | 1 | | 1 |
| **Travel time to visit the neurologist based in the general neurology clinic (one way)** | | | | | | | | |
|  | **Patients who reported never attending a SAC (N=22)** | | | | **Patients who reported attending a SAC currently (N=75)** | | | |
|  | **N** | | **%** | | **N** | | **%** | |
| Less than 1 hour | 10 | | 40 | | 50 | | 67 | |
| 1 to 2 hours | 8 | | 32 | | 15 | | 20 | |
| 2 to 3 hours | 1 | | 4 | | 2 | | 3 | |
| 3 to 4 hours | 2 | | 8 | | 1 | | 1 | |
| More than 4 hours | 1 | | 4 | | 2 | | 3 | |
| Not applicable | 1 | | 4 | | 7 | | 7 | |
| Unsure | 2 | | 8 | |  | |  | |
| **Mode of transport mainly used to visit the neurologist based in the general neurology clinic** | | | | | | | | |
|  | **Patients who reported never attending a SAC (N=26)** | | | | **Patients who reported attending a SAC currently (N=72)** | | | |
| Bus |  | |  | | 2 | | 3 | |
| Car | 18 | | 69 | | 62 | | 86 | |
| Taxi | 2 | | 7 | | 1 | | 1 | |
| Train | 1 | | 4 | | 3 | | 4 | |
| Other | 5 | | 19 | | 3 | | 4 | |
| Unsure |  | |  | | 1 | | 1 | |

SAC, specialist ataxia centre; N, number of participants who responded to that question.

Supplementary Table 19: Living location of participants


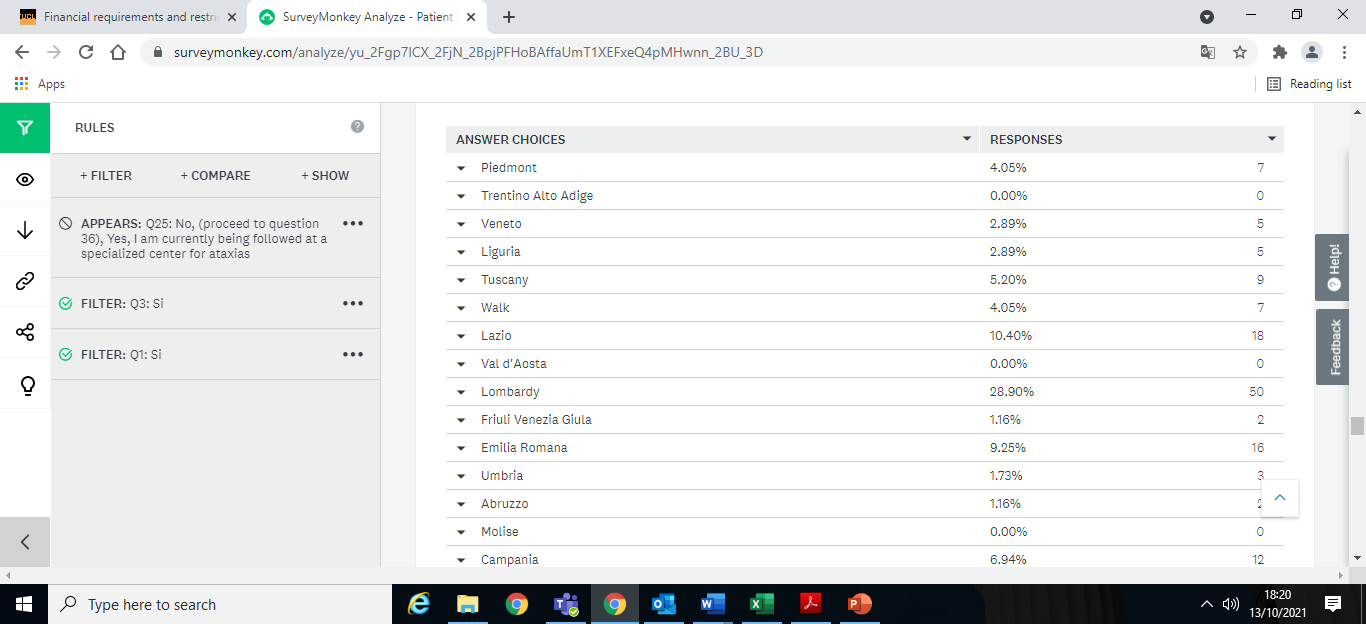

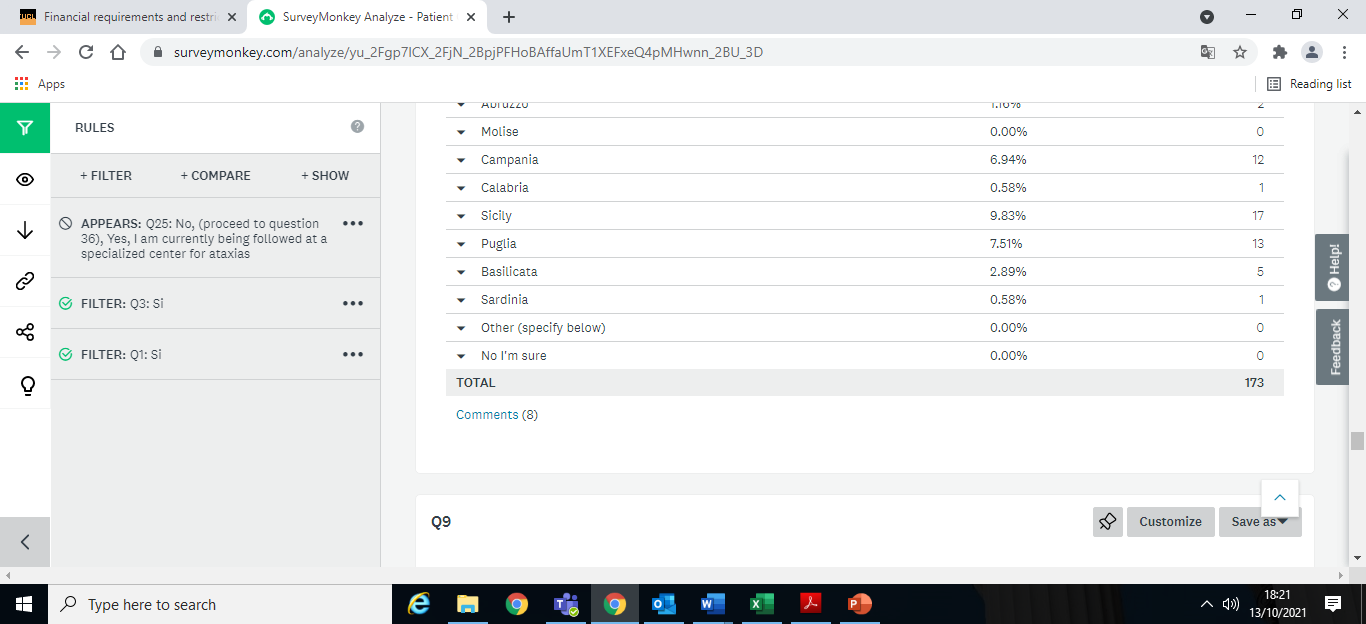

Supplement: Supplementary file 2 — Supplementary file2 (DOCX 5053 KB) [file 10072_2026_9150_MOESM2_ESM.docx]
